# Supplementary material for: Uncontrolled hypertension among adult hypertensive patients in Addis Ababa public hospitals: A cross-sectional study of prevalence and associated factors
Source: PLoS One. 2024 Dec 30;19(12):e0316461. doi: 10.1371/journal.pone.0316461 (PMC11684623; doi:10.1371/journal.pone.0316461)
Supplement: Attachment — Submitted filename: Comment to the author-PONE-D-24-37212.pdf [file pone.0316461.s001.pdf]

<table>
<tr><td>Peer Evaluation for the Authors:</td><td>Original Submission
Prevalence and associated factors of uncontrolled hypertension among adult hypertensive patients at public hospitals in Addis

Authors: Asmamaw Deguale Worku, MPH
Asinake Wudu Gessese, MPH</td></tr>
<tr><td colspan="2">First and foremost, thank you for the opportunity to review your manuscript.

This feedback is intended to assist the authors in refining their work to meet high academic standards and ensure that their findings contribute effectively to the existing body of knowledge on uncontrolled hypertension.

I look forward to the authors' revisions which incorporate these suggestions to strengthen the methodological rigor and ethical transparency of the study.

These suggestions are intended to improve the clarity, ethical integrity, and methodological transparency of the manuscript, thus strengthening its overall scientific contribution.

By addressing the following aspects, the authors can ensure that the study is scientifically rigorous and ethically compliant, providing transparency in both variable measurement and data protection.</td></tr>
<tr><td>Section</td><td>Comments</td></tr>
<tr><td>Background:

Risk factors: Personal history of CVD (myocardial infarction, heart failure [HF], stroke, transient ischemic attacks [TIA], diabetes, dyslipidemia, chronic kidney disease [CKD], smoking status, diet, alcohol intake, physical activity, psychosocial aspects, history of depression). Family history of hypertension, premature CVD, (familial) hypercholesterolemia, diabetes.

CVD: More than 50% of hypertensive patients have additional cardiovascular risk factors.[28,29] The most common additional risk factors are diabetes (15%–20%), lipid disorders (elevated low-density lipoprotein-cholesterol [LDL-C] and triglycerides [30%]), overweight-obesity (40%), hyperuricemia (25%) and metabolic syndrome (40%), as well as unhealthy lifestyle habits (eg, smoking, high alcohol intake, sedentary lifestyle).[28–30] The presence of one or more additional cardiovascular risk factors proportionally increases the risk of coronary, cerebrovascular, and renal diseases in hypertensive patients.[1] Other Risk Factors: Age (>65 years), sex (male>female), heart rate (>80 beats/min), increased body weight,</td><td>Background:

Reorganization: The background section would benefit from reorganization to ensure a logical flow of ideas. Specifically:

Lines 46-59, which provide a clear definition of hypertension, are well-presented and should remain as is.

Lines 61-71 discuss risk factors for hypertension. More details could be added to explain the pathophysiology behind these risk factors, which may offer readers a deeper understanding.

In lines 73-87, while the authors mention methods to prevent and control HTN, there is a need to enhance this section by referencing established guidelines such as the American Heart Association (AHA) or the Eighth Joint National Committee (JNC-8). Prevention and control strategies should be categorized more clearly, e.g., (1) Lifestyle Modifications, (2) Pharmacological Treatments, and (3) Adherence to Antihypertensive Treatment. This will offer a more structured and evidence-based approach, emphasizing treatment adherence, a key issue in uncontrolled hypertension.

Research Questions: There is room to better link the background to the two primary research questions:</td></tr>
</table>

diabetes, high LDL-C/triglyceride, family history of CVD, family history of hypertension, early-onset menopause, smoking habits, psychosocial or socioeconomic factors. **HMOD:** LVH (LVH with ECG), moderate-severe CKD (CKD; eGFR <60 ml/min/1.73m^2), any other available measure of organ damage. **Disease:** previous coronary heart disease (CHD), HF, stroke, peripheral vascular disease, atrial fibrillation, CKD stage 3+. (https://www.ahajournals.org/doi/<br><br>10.1161/HYPERTENSIONAHA.120.15026) or https://www.umpquahealth.com/wp-content/uploads/2019/03/jnc-8-hypertension-guideline-algorithm.pdf	What is the prevalence of uncontrolled hypertension?<br><br>What factors are associated with uncontrolled hypertension?<br><br>In lines 89-100, the global and regional epidemiology of hypertension is discussed. This section could be more concise and aligned with the study's research questions. It would be beneficial to frame the epidemiological data in a way that transitions smoothly to the study's objectives, focusing on the prevalence of uncontrolled hypertension.<br><br>Literature Review: Conciseness and Relevance: Between lines 89-140, the manuscript discusses previous studies in great depth. However, the defense of prior research becomes overly detailed. It is recommended to streamline this section by summarizing key studies that directly relate to the risk factors and treatment strategies for hypertension, particularly uncontrolled HTN. Moreover, the review should be more focused on aligning literature with the study's objectives, especially regarding the unique conditions of Ethiopia.<br><br>Gap Identification: In lines 142-154, the authors discuss the gap in research and knowledge in the Ethiopian context. This section effectively highlights the rationale for the study. However, it would be beneficial to conclude this section by reiterating the specific factors of uncontrolled HTN that this study aims to address. Doing so will reinforce the significance of the research in contributing new insights to existing knowledge.
Methods	Section: Methods<br><br>The setting, population, and eligibility criteria are well explained, and the Sample Size Determination and Sampling Procedures are presented clearly, particularly through the use of Figure 1. However, several aspects of the methodology require further elaboration to enhance the clarity and transparency of the study's design and procedures.<br><br>Sample Size Determination and Sampling Procedures: Participant Recruitment: While the authors mention that data were collected using systematic random sampling (with every eighth participant selected), it is unclear how the study ensured that these specific participants attended their appointments on time. The manuscript should provide further detail on how the authors managed participant scheduling and attendance. For instance, how did the team handle instances where selected participants were unavailable or did not show up? Were participants

	rescheduled, or were new participants chosen? A clear explanation of these procedures is crucial to understanding how bias or sampling errors were avoided.<br><br>Ethical Considerations: Vulnerable Populations: The data collection was conducted by four BSc nurses under close supervision by two health officers and the principal investigator. While this approach ensures professionalism, there is a potential ethical concern due to the involvement of health providers directly in the data collection from patients, who may feel obliged to participate. It is recommended that the authors clarify how they addressed these potential ethical vulnerabilities, particularly regarding patient consent and the possibility of coercion. Was there a mechanism in place to ensure that participants felt free to refuse participation without consequence? Did the nurses or investigators receive specific ethical training to handle such situations?<br><br>Blood Pressure Assessment: Calibration and Standardization: The authors indicate that blood pressure was measured using an analog BP sphygmomanometer and BP cuff, assessing the brachial artery. However, it is essential to detail the steps taken to ensure the accuracy of these measurements. The manuscript should state how the equipment was calibrated and standardized before and during the study. If no calibration process was used, the authors should justify this omission, or if it was performed, they should provide information on how frequently it was conducted and whether standardized protocols (e.g., WHO or AHA guidelines) were followed. Calibration procedures are critical for ensuring the validity and reliability of blood pressure measurements, which are key outcomes of the study.<br><br>Section: Variables and Ethical Considerations<br><br>Independent and Dependent Variables: Clarification of Variables: The authors should explicitly mention all independent and dependent variables within the background section of the manuscript. This will provide the reader with a clear understanding of how each variable relates to the study's objectives and its relevance to the research questions. For instance, how are physical activity (PA) and other health behaviors being measured? Are there validated tools or questionnaires used for these measurements? The authors should reference appropriate tools or methods used for measurement.

	Physical Activity (PA): In the second research question (e.g., "How many of the past seven days did you perform specific exercise activities such as swimming, walking, or biking?"), the authors should include a detailed explanation of how responses to this question were scored and interpreted. If PA is being measured through self-reported questionnaires, the validity and reliability of these tools should be referenced, and the scoring method (e.g., how points are assigned based on frequency or type of activity) should be clearly described.<br><br>Awareness of hypertension-related complications was assessed using a single question with multiple response items. Participants were classified into two categories:<br><br>Aware: Participants who were knowledgeable about at least two hypertension-related complications.<br><br>Not Aware: Participants who either did not know of any hypertension-related complications or were aware of only one complication.<br><br>This assessment was conducted using the Hypertension Self-Care Activity Level Effects (H-SCALE) tool from 9. Tesfaye B,. The authors should reference the following study for the validation and prevalence of hypertension self-care activities: Warren-Findlow J, Seymour RB. Prevalence rates of hypertension self-care activities among African Americans. J Natl Med Assoc. 2011;103(6):503. This reference will provide a robust foundation for the methodology used in measuring self-care activities and awareness in the context of hypertension.<br><br>Ethical Considerations Regarding Clinical Variables:<br><br>Patient Clinical Variables: The manuscript includes patient clinical variables, such as health data related to hypertension. It is crucial for the authors to clarify how they obtained this information. Did the data come from Electronic Health Records (EHR) or Personal Health Records (PHR)? If so, the authors should detail the procedures followed to access this data, ensuring that patient consent and confidentiality were fully protected.<br><br>Ethical Compliance (PDPA): To avoid potential breaches of data privacy, the authors should ensure that their procedures comply with relevant data protection laws, such as the Personal Data Protection Act (PDPA) or other applicable regulations. Explicit mention of how patient confidentiality was preserved is necessary, including anonymization or de-

	identification of clinical data. The manuscript should also describe how patient consent was obtained for using their clinical data and clarify whether any additional ethical approvals were obtained from institutional review boards (IRBs) or ethics committees.
Results	The findings is demonstrated follow the objective and research question and well organized.
Discussion<br><br><br><br><br><br>Conclusion	Discussion and Conclusion (Suggestions for Improvement):<br><br>The authors have thoroughly discussed the study's characteristics (Table 1, Table 2, and Table 3), along with the determinants of factors associated with uncontrolled hypertension (Table 4). However, the discussion could be improved by ensuring a stronger and more coherent link between the findings presented in the tables and the overarching discussion throughout the manuscript.<br><br>Study Findings: The key finding of the study is that the odds of having uncontrolled hypertension were 5.21 times greater for those who did not adhere to antihypertensive medication compared to those who did. This important insight should be emphasized more clearly in both the discussion and the conclusion sections.<br><br>Concise Conclusion: The primary conclusion drawn from the study is that non-adherence to antihypertensive medication is a significant predictor of uncontrolled hypertension. However, while the recommendation to healthcare professionals is appropriate, the conclusion could be extended to propose more comprehensive solutions beyond the current suggestions of weight reduction, health education, and control of comorbidities.<br><br>Extended Solutions: for instance 1) Improving Medication Adherence: The findings strongly suggest that targeted interventions to improve adherence to antihypertensive medication should be prioritized. 2) This could involve:Implementing structured follow-up and reminder systems for patients.3) Enhancing patient education on the importance of medication adherence and its long-term benefits.4)Utilizing digital health tools, such as mobile apps, to track adherence and offer real-time support.<br><br>Multidisciplinary Care Models: Given the multifactorial nature of hypertension, a team-based care approach involving physicians, nurses, pharmacists, and community health workers should be advocated. This would help to

	monitor not only blood pressure but also modifiable lifestyle factors more comprehensively.<br><br>Patient-Centered Approaches: Empowering patients to take an active role in managing their hypertension could improve outcomes. This can be achieved by involving them in decision-making regarding their treatment plans and offering personalized care based on individual risk factors.<br><br>Policy Advocacy: Beyond healthcare professionals, the study suggests that broader community-based and policy-driven initiatives are necessary. This could involve:Implementing national campaigns aimed at increasing awareness about the importance of medication adherence. Introducing financial incentives or subsidies for antihypertensive medications to improve access for low-income populations. By proposing these comprehensive, patient-centered, and policy-driven solutions, the conclusion would not only reflect the study's findings but also offer practical steps that could be taken to address the challenge of uncontrolled hypertension.
