# Supplementary material for: Uncontrolled hypertension among adult hypertensive patients in Addis Ababa public hospitals: A cross-sectional study of prevalence and associated factors
Source: PLoS One. 2024 Dec 30;19(12):e0316461. doi: 10.1371/journal.pone.0316461 (PMC11684623; doi:10.1371/journal.pone.0316461)
Supplement: Attachment — Submitted filename: Comment to -PONE-D-24-37212R1.pdf [file pone.0316461.s003.pdf]

<table>
<tr><td>Peer Evaluation for the Authors:</td><td>PONE-D-24-37212R1
Uncontrolled Hypertension in Adult Patients in Addis Ababa Public Hospitals: Prevalence and Associated Factors
PLOS ONE
Authors: Asmamaw Deguale Worku, MPH
Asinake Wudu Gessese, MPH</td></tr>
<tr><td colspan="2">Reviewer Acknowledgment:

Thank you for the opportunity to review this revised manuscript titled "Uncontrolled Hypertension in Adult Patients in Addis Ababa Public Hospitals: Prevalence and Associated Factors." I appreciate the authors’ responsiveness and diligence in addressing all previous comments.

The authors have thoroughly addressed each suggestion, resulting in a clearer and more comprehensive presentation of the study. I am pleased with the revisions, which have strengthened the manuscript, and I have some of comments to considerate below.</td></tr>
<tr><td>Title</td><td>The title change enhances clarity and aligns well with the manuscript's focus.</td></tr>
<tr><td>Abstract</td><td>The abstract is significantly improved. However, the conclusion could better emphasize the most critical risk factors for uncontrolled hypertension in this setting. Key factors to highlight include non-adherence to antihypertensive medications (OR 5.21), use of multiple medications (OR 3.10), lack of physical exercise (OR 2.84), and low adherence to low-salt and DASH diets (OR 2.74). To address these, focus on enhancing patient education on medication adherence, promoting lifestyle changes, and leveraging digital health tools, like mobile apps, for real-time support and adherence tracking.</td></tr>
<tr><td>Background
Methods
Results
Discussion</td><td>Thank you for the thoughtful revisions. The background, methods, results, and discussion sections are much improved and offer a clearer presentation of the study compared to the previous version.</td></tr>
<tr><td>Conclusion</td><td>However, the conclusion remains overly lengthy. I recommend condensing and refining it to ensure a more concise and impactful summary of the key findings and recommendations.

From the previous comments about Recommendations include implementing follow-up and reminder systems, enhancing patient education on adherence benefits, and using digital health tools to support real-time tracking.

Also in line 628 -640 are the recommendation as a Multi-disciplinary Care Models, Patient-Centered Approaches, Policy Advocacy , the authors included in this part and might make it concise and decrease the duplicate in line 641-649,

It would be beneficial to delete lines 651-657, as they introduce additional recommendations that do not directly align with the main focus of the study's findings or with the core recommendations on improving hypertension control. This section introduces new topics, such as mass-sporting activities and specific local projects, which may dilute the impact of the more targeted suggestions on</td></tr>
</table>

	medication adherence, lifestyle changes, and digital tools. Additionally, the recommendation for future studies, though useful, may not be necessary in the conclusion if it has already been mentioned in the discussion.<br><br>In summary, removing these lines would help make the conclusion more concise and focused, allowing the key findings and primary recommendations to stand out more clearly.
