# Supplementary material for: Uncontrolled hypertension among adult hypertensive patients in Addis Ababa public hospitals: A cross-sectional study of prevalence and associated factors
Source: PLoS One. 2024 Dec 30;19(12):e0316461. doi: 10.1371/journal.pone.0316461 (PMC11684623; doi:10.1371/journal.pone.0316461)
Supplement: Attachment — Submitted filename: Response to Reviewers.docx [file pone.0316461.s002.docx]

**Dear editor and reviewers,**

Response: The authors are thankful for the detailed and specific comments on the manuscript. As it is a scientific study, we strongly believe that this will assist to meet high academic standards and ensure that our findings contribute effectively to the existing body of knowledge on uncontrolled hypertension. Most comments focused on the abstract, introduction, methodology, result, discussion and conclusion section as well as journal requirements. Therefore, detailed and extensive revision is made regarding abstract, introduction, methodology, result, discussion and conclusion section as well as journal requirements. With all this revision, we believe that the study will meet high academic standards and contribute to existing body of knowledge in the study area. Specific responses and revisions made are provided below and highlighted in the main document. Hopefully, the editor will consider the revised manuscript for publication.

**Uncontrolled Hypertension in Adult Patients in Addis Ababa Public Hospitals: Prevalence and Associated Factors."**

To: editor,

Above all, we thank you for your constructive suggestions and comments on this manuscript that would improve the substance and content of the study. We considered each comment and clarification question of editor on the manuscript thoroughly. Our point-by-point responses for each comment and question are described in detail on the following pages. All newly modified changes were highlighted within the main manuscript. Please see the red font color in the clean revised manuscript.

Editor Comments and Suggestions

1. If you would like to make changes to your financial disclosure, please include your updated statement in your cover letter

Response: Thanks a lot for your suggestions! This revised manuscript has no any change of financial disclosure.

2. Guidelines for resubmitting your figure files are available below the reviewer comments at the end of this letter. While revising your submission, please upload your figure files to the Preflight Analysis and Conversion Engine (PACE) digital diagnostic tool

Response: Thanks a lot for your eagle eyes! The editor is correct. We are very grateful for this implicit comment and suggestion. Thank you so much for such valuable intellectual input. Now, based on your suggestions and comments, we have thoroughly, uploaded in to the Preflight Analysis and Conversion Engine (PACE) digital diagnostic tool to change the figure based on the PLOS ONE submission guideline. The newly modified change was highlighted with a red font color in the cleanly revised manuscript.

3. If applicable, we recommend that you deposit your laboratory protocols in [protocols.io](http://protocols.io/) to enhance the reproducibility of your results. Protocols.io assigns your protocol its own identifier (DOI) so that it can be cited independently in the future. Additionally, PLOS ONE offers an option for publishing peer-reviewed Lab Protocol articles, which describe protocols hosted on [protocols.io](http://protocols.io/).

Response: Thank you very much. The editor is perfect, but this study is not laboratory protocol.

[4. When submitting your revision, we need you to address these additional requirements. Please ensure that your manuscript meets PLOS ONE's style requirements, including those for file naming](http://c05y1x9s.r.us-east-2.awstrack.me/L0/file:%2F%2F%2FC:%2FUsers%2Fe754871%2FDesktop%2FPRTC%2FPRTC%25202024%2FSeptember%25202024%2FSeptember%252027,%25202024%2FPONE-D-24-37212%2FJournal%2520requirements:%2520%250b%250bWhen%2520submitting%2520your%2520revision,%2520we%2520need%2520you%2520to%2520address%2520these%2520additional%2520requirements.%2520%250b%250b1.%2520Please%2520ensure%2520that%2520your%2520manuscript%2520meets%2520PLOS%2520ONE's%2520style%2520requirements,%2520including%2520those%2520for%2520file%2520naming.%2520The%2520PLOS%2520ONE%2520style%2520templates%2520can%2520be%2520found%2520at%2520https:%2Fjournals.plos.org%2Fplosone%2Fs%2Ffile%253fid=wjVg%2FPLOSOne_formatting_sample_main_body.pdf%2520and/1/010f0192334ede1b-683b53d2-6771-4e17-8ad3-67825063c68d-000000/VZXmBsCBSTnF91LdE7c4UiWgDhk=178)

Response: Thanks a lot for your eagle eyes! The editor is correct. We are very grateful for this implicit comment and suggestion. Thank you so much for such valuable intellectual input. Now, based on your suggestions and comments, we have thoroughly revised and edited all parts of our manuscript depending on PLOS ONE's style requirements including file naming. The newly modified change was highlighted with a red font color in the cleanly revised manuscript.

5. In the online submission form, you indicated that “The datasets used and/or analyzed during the current study are available from the corresponding author upon reasonable request.”

All PLOS journals now require all data underlying the findings described in their manuscript to be freely available to other researchers, either 1. In a public repository, 2. Within the manuscript itself, or 3. Uploaded as supplementary information. This policy applies to all data except where public deposition would breach compliance with the protocol approved by your research ethics board. If your data cannot be made publicly available for ethical or legal reasons (e.g., public availability would compromise patient privacy), please explain your reasons on resubmission and your exemption request will be escalated for approval.

Response: Thanks a lot for your eagle eyes! The editor is perfect. We are very grateful for this implicit comment and suggestion. Thank you so much for such valuable intellectual input. Now, based on your suggestions and comments, we have thoroughly revised and edited all parts of our manuscript. All necessary materials used and/or analyzed during the current study were included in the manuscript and the online submission form will be corrected during submission.

6. Your ethics statement should only appear in the Methods section of your manuscript. If your ethics statement is written in any section besides the Methods, please move it to the Methods section and delete it from any other section. Please ensure that your ethics statement is included in your manuscript, as the ethics statement entered into the online submission form will not be published alongside your manuscript.

Response: Thank you very much. The editor is perfect. We are grateful for your robust and rigorous comments and suggestions. Hence, the authors absolutely share the concerns and comments of editor. Thus, now with great appreciation, the authors considered your valid concern and explicitly incorporation of the ethics statement in the methods section. The newly modified change was highlighted with a red font color in the cleanly revised manuscript.

7. Please include your tables as part of your main manuscript and remove the individual files. Please note that supplementary tables (should remain/ be uploaded) as separate "supporting information" files"

Response: Definitely! The editor is correct. We are grateful for this implicit comment and suggestion. The authors critically reviewed this vital comment and corrected the necessary modification as per your robust and critical comments. The tables were included in the main manuscript and removed the individual files. The newly modified change was highlighted with red font color in the clean revised main manuscript

8. We note that there is identifying data in the Supporting Information file <Table 1.docx and Table 4.docx>. Due to the inclusion of these potentially identifying data, we have removed this file from your file inventory. Prior to sharing human research participant data, authors should consult with an ethics committee to ensure data are shared in accordance with participant consent and all applicable local laws. Data sharing should never compromise participant privacy. It is therefore not appropriate to publicly share personally identifiable data on human research participants. The following are examples of data that should not be shared: -Name, initials, physical address -Ages more specific than whole numbers -Internet protocol (IP) address -Specific dates (birth dates, death dates, examination dates, etc.) -Contact information such as phone number or email address -Location data -ID numbers that seem specific (long numbers, include initials, titled “Hospital ID”) rather than random (small numbers in numerical order) Data that are not directly identifying may also be inappropriate to share, as in combination they can become identifying. For example, data collected from a small group of participants, vulnerable populations, or private groups should not be shared if they involve indirect identifiers (such as sex, ethnicity, location, etc.) that may risk the identification of study participants. Please remove or anonymize all personal information (<specific identifying information in file to be removed>), ensure that the data shared are in accordance with participant consent, and re-upload a fully anonymized data set. Please note that spreadsheet columns with personal information must be removed and not hidden as all hidden columns will appear in the published file.

Response: Definitely! The editor is correct. We are grateful for this implicit comment and suggestion. The authors critically reviewed this vital comment and there is no personally identifiable data that data sharing should compromise participant privacy.

9. Additional Editor Comments:

Although at times the writing is strong and clear, throughout most of the document the writing is circuitous, unclear and contains grammatical errors that obscure meaning. Recommend the entire paper be edited for conciseness, clarity and grammar before re-submission.

Response: Thanks a lot for your eagle eyes! The editor is correct. We are very grateful for this implicit comment and suggestion. Thank you so much for such valuable intellectual input. Now, based on your suggestions and comments, we have thoroughly modified, edited and revised the entire document of the manuscript. The newly modified change was highlighted with a red font color in the cleanly revised manuscript.

10. In Abstract add the full form of DASH when its first used.

Response: Definitely! The editor is correct. We are grateful for this implicit comment and suggestion. The authors critically reviewed this vital comment and corrected the necessary modification as per your robust and critical comments. The newly modified change was highlighted with red font color in the clean revised main manuscript.

11. The introduction is a major issue. As the first part of the article that a potential reader reads, it should be attractive and easy to read. The authors can make it concise. A lot of information such as hypertension definition, and types of anti-hypertensive medications can be removed. Definitions of uncontrolled hypertension should be moved to methods. The details of previous studies should be presented in very brief. Are there any current interventions in the study area for uncontrolled hypertension (Authors can add a brief paragraph). I propose the authors use, consult, and add the following reference in the manuscript.

Response: Thanks a lot for your eagle eyes! The editor is perfect. We are grateful for this implicit comment and suggestion. The authors critically reviewed this vital comment and removed hypertension definition, and types of anti-hypertensive medications in the introduction section, and moved definitions of uncontrolled hypertension to the method parts as per your robust and critical comments. The authors also referenced the recommended paper to the manuscript. The newly modified change was highlighted with red font color in the clean revised main manuscript.

12. Lines 177-178: Difficult to understand. Please rephrase

Response: Thanks a lot for your eagle eyes! The editor is correct. We are very grateful for this implicit comment and suggestion. Thank you so much for such valuable intellectual input. Now, based on your suggestions and comments, we have thoroughly, revised, paraphrased and edited the manuscript. The newly modified change was highlighted with red font color in the clean revised manuscript.

13. Line 187: Please add a space between citation and text. Please follow this through all manuscript.

Response: Definitely! Many thanks for it. We are grateful for this crucial remark. Editor, you are flawless. This significant observation of highlights was thoroughly addressed in the updated paper. The newly modified change was highlighted with red font color in the clean revised manuscript.

14. Line 209: Please merge both citations. Please follow this through all manuscript.

Response: Definitely! Thank you very much. We appreciate this important observation. Dear editor, you are perfect. We made significant revisions on citations and merged it throughout all documents. Please refer to the revised red font color in the cleanly revised manuscript.

15. Line 253: Uncontrolled hypertension definition: Please revise as currently patient with uncontrolled hypertension not uncontrolled hypertension.

Response: Thanks a lot for your eagle eyes! The editor is correct. We are very grateful for this implicit comment and suggestion. Thank you so much for such valuable intellectual input. Now, based on your suggestions and comments, we have thoroughly revised the manuscripts as well edited all parts of our manuscript. The newly modified change was highlighted with a red font color in the cleanly revised manuscript.

16. Lines 257-260: Please add references to medication adherence and other independent variables where applicable.

Response: Thank you so much for pointing this out! Many thanks for it. We are grateful for this crucial remark. Editor, you are flawless. This significant observation was thoroughly addressed and cited in the updated paper. Please refer to the neatly corrected content and the updated red font color.

17. Lines 389-391: Difficult to understand. Please rephrase

Response: Definitely! Thank you very much. We are very grateful for this implicit comment and suggestion. Thank you so much for such valuable intellectual input. Now, based on your suggestions and comments, we have thoroughly, revised, paraphrased and edited the manuscript. The newly modified change was highlighted with red font color in the clean revised manuscript.

18. The authors can summarize the prevalence of uncontrolled hypertension when compared with other LMICs. At current form it is difficult to understand. They can revise us the prevalence of uncontrolled hypertension in LMICs ranges from

Response: Thank you so much for such valuable intellectual input. The editor is perfect. We are very grateful for this implicit comment and suggestion. Thank you so much for such valuable intellectual input. The authors critically considered this valid input and revised it. The newly modified change was highlighted with a red font color in the cleanly revised manuscript.

19. Recommend that authors re-write the discussion by condensing the information into well-formed paragraphs that link together to form a story of what they recommend from each of the key observations they have made.

Response: Thank you, a lot, for pointing out this!  I am really grateful for your insightful comments. The author fully offers the utterly shares the comments and suggestion, which significantly improve the manuscript's quality. The newly modified change was highlighted with red font color in the clean revised manuscript.

20. Conclusions would benefit from more concise language and clear, specific study-based recommenda6ons.

Response: Thank you so much for pointing this out. The editor is flawless. We greatly appreciate your implied observation and recommendation. We critically revised and addressed this important observation in the revised manuscript. The newly modified change was highlighted with red font color in the clean revised manuscript.

21. Please include Tables in the main manuscript file.

Response: Definitely! The editor is correct. We are grateful for this implicit comment and suggestion. The authors critically reviewed and included tables in the main manuscript. The newly modified change was highlighted with red font color in the clean revised manuscript.

**To Reviewers’**

Above all, we thank you for your constructive comments and helpful suggestions that helped us to improve and enrich our study. We considered each comment and clarification question of reviewers on the manuscript thoroughly. Our point-by-point responses for each comment and question are described in detail on the following pages. All newly modified changes were highlighted with red font color in the main manuscript. Please see the red font color in the clean revised manuscript.

Reviewer #1

22. Background: Reorganization: The background section would benefit from reorganization to ensure a logical flow of ideas. Specifically: Lines 46-59, which provide a clear definition of hypertension, are well-presented and should remain as is.

Response: Thank you so much! Many thanks for it. The authors would also thankful for your effort and dedication to improve the quality of this study.

23. Lines 61-71 discuss risk factors for hypertension. More details could be added to explain the pathophysiology behind these risk factors, which may offer readers a deeper understanding.

Response: Thank you so much to pointing this out. The reviewer is perfect. We are very grateful for this implicit comment and suggestion. Authors critically considered this valid input. The authors critically reviewed this vital comment and corrected the necessary modification as per your robust and critical comments. The newly modified change was highlighted with red font color in the clean revised main manuscript.

24. In lines 73-87, while the authors mention methods to prevent and control HTN, there is a need to enhance this section by referencing established guidelines such as the American Heart Association (AHA) or the Eighth Joint National Committee (JNC-8). Prevention and control strategies should be categorized more clearly, e.g., (1) Lifestyle Modifications, (2) Pharmacological Treatments, and (3) Adherence to Antihypertensive Treatment. This will offer a more structured and evidence-based approach, emphasizing treatment adherence, a key issue in uncontrolled hypertension.

Response: Thank you so much for such valuable intellectual input. The reviewer is perfect. We are very grateful for this implicit comment and suggestion. Thank you so much for such valuable intellectual input. The authors critically considered this valid input. The newly modified change was highlighted with a red font color in the cleanly revised manuscript.

25. Research Questions: There is room to better link the background to the two primary research questions: What is the prevalence of uncontrolled hypertension? What factors are associated with uncontrolled hypertension?

Response: Thanks a lot for your eagle eyes!  The reviewer is correct. We are very grateful for this implicit comment and suggestion. Thank you so much for such a valuable intellectual input. The authors critically considered this valid input. The newly modified change was highlighted with a red font color in the cleanly revised manuscript.

26. In lines 89-100, the global and regional epidemiology of hypertension is discussed. This section could be more concise and aligned with the study's research questions. It would be beneficial to frame the epidemiological data in a way that transitions smoothly to the study's objectives, focusing on the prevalence of uncontrolled hypertension.

Response: Thank you, a lot, for pointing out this!  I am really grateful for your insightful comments. The author fully offers the utterly shares the comments and suggestion, which significantly improve the manuscript's quality. The newly modified change was highlighted with red font color in the clean revised manuscript

27. Literature Review: Conciseness and Relevance: Between lines 89-140, the manuscript discusses previous studies in great depth. However, the defense of prior research becomes overly detailed. It is recommended to streamline this section by summarizing key studies that directly relate to the risk factors and treatment strategies for hypertension, particularly uncontrolled HTN. Moreover, the review should be more focused on aligning literature with the study’s objectives, especially regarding the unique conditions of Ethiopia.

Response: Thank you so much for pointing this out. The reviewer is flawless. We greatly appreciate your implied observation and recommendation. The authors include relevant literature, summarize and revised the manuscript particularly focusing on uncontrolled HTN and study objectives. The newly modified change was highlighted with red font color in the clean revised manuscript.

28. Gap Identification: In lines 142-154, the authors discuss the gap in research and knowledge in the Ethiopian context. This section effectively highlights the rationale for the study. However, it would be beneficial to conclude this section by reiterating the specific factors of uncontrolled HTN that this study aims to address. Doing so will reinforce the significance of the research in contributing new insights to existing knowledge.

Response: Definitely! Thank you very much. We appreciate this important observation. Dear reviewer, you are perfect. We critically revised and addressed this important observation in the revised manuscript. Please refer to the revised newly modified manuscript.

29. Section: Methods The setting, population, and eligibility criteria are well explained, and the Sample Size Determination and Sampling Procedures are presented clearly, particularly through the use of Figure 1. However, several aspects of the methodology require further elaboration to enhance the clarity and transparency of the study’s design and procedures.

Response: Thank you, a lot, for pointing out this!  Thank you so much for such valuable intellectual input. The Author utterly shares the comments and suggestion which immensely enhance the manuscript. The newly modified change was highlighted with red font color in the clean revised manuscript

30. Sample Size Determination and Sampling Procedures: Participant Recruitment: While the authors mention that data were collected using systematic random sampling (with every eighth participant selected), it is unclear how the study ensured that these specific participants attended their appointments on time. The manuscript should provide further detail on how the authors managed participant scheduling and attendance. For instance, how did the team handle instances where selected participants were unavailable or did not show up? Were participants rescheduled, or were new participants chosen? A clear explanation of these procedures is crucial to understanding how bias or sampling errors were avoided.

Response: Thank you so much to pointing this out. The reviewer is perfect. We are very grateful for this implicit comment and suggestion. Authors critically considered this valid input. Dear respected reviewer, we used patients’ visit as a sampling frame for our study. For instance, we recruited patients based on their visit at hypertension follow-up clinic, based on our inclusion criteria. If we had used patient’s register or patients’ appointment register, those patients who missed their appointment (follow-up) would have been an issue. Otherwise, in our case it is not an issue. We tried to incorporate your comment in the revised manuscript.

31. Ethical Considerations: Vulnerable Populations: The data collection was conducted by four BSc nurses under close supervision by two health officers and the principal investigator. While this approach ensures professionalism, there is a potential ethical concern due to the involvement of health providers directly in the data collection from patients, who may feel obliged to participate. It is recommended that the authors clarify how they addressed these potential ethical vulnerabilities, particularly regarding patient consent and the possibility of coercion. Was there a mechanism in place to ensure that participants felt free to refuse participation without consequence? Did the nurses or investigators receive specific ethical training to handle such situations?

Response: Thank you very much. The reviewer is perfect. We are grateful for your robust and rigorous comments and suggestions. Hence, the author utterly shares the concerns and comments of the reviewer. Thus, now, with great appreciation, the authors have considered your valid concern. It is true that involvement of the healthcare providers could make patients feel obliged to be participated in the study. However, the data collectors were given two days training on the study's objective, relevance, and confidentiality of information, respondents' rights, informed consent, and interview techniques. The newly modified change was highlighted with red font color in the clean revised manuscript.

32. Blood Pressure Assessment: Calibration and Standardization: The authors indicate that blood pressure was measured using an analog BP sphygmomanometer and BP cuff, assessing the brachial artery. However, it is essential to detail the steps taken to ensure the accuracy of these measurements. The manuscript should state how the equipment was calibrated and standardized before and during the study. If no calibration process was used, the authors should justify this omission, or if it was performed, they should provide information on how frequently it was conducted and whether standardized protocols (e.g., WHO or AHA guidelines) were followed. Calibration procedures are critical for ensuring the validity and reliability of blood pressure measurements, which are key outcomes of the study.

Response: Thanks a lot for your eagle eyes! The reviewer is correct. We are very grateful for this implicit comment and suggestion. The sphygmomanometer was calibrated by biomedical engineers before the actual data collection to ensure the reliability and validity of measurement. Additionally, the measurement was cross-checked with the other sphygmomanometers in the hospitals prior to measurement to check for the discrepancies. The newly modified changes were highlighted with a red font color in the cleanly revised manuscript. Thank you so much for such valuable intellectual input.

33. Section: Variables and Ethical Considerations Independent and Dependent Variables: Clarification of Variables: The authors should explicitly mention all independent and dependent variables within the background section of the manuscript. This will provide the reader with a clear understanding of how each variable relates to the study's objectives and its relevance to the research questions. For instance, how are physical activity (PA) and other health behaviors being measured? Are there validated tools or questionnaires used for these measurements? The authors should reference appropriate tools or methods used for measurement.

Response: Definitely! The reviewer is correct. We are grateful for this implicit comment and suggestion. The authors critically reviewed this vital comment and we use a validated tool and referenced it as per your robust and critical comments. Authors critically considered this valid input. The newly modified change was highlighted with a red font color in the cleanly revised main manuscript.

34. Physical Activity (PA): In the second research question (e.g., "How many of the past seven days did you perform specific exercise activities such as swimming, walking, or biking?"), the authors should include a detailed explanation of how responses to this question were scored and interpreted. If PA is being measured through self-reported questionnaires, the validity and reliability of these tools should be referenced, and the scoring method (e.g., how points are assigned based on frequency or type of activity) should be clearly described.

Response: Response: Definitely! The reviewer is correct. We are grateful for this implicit comment and suggestion. The authors critically reviewed this vital comment and we use a validated tool and referenced it as per your robust and critical comments. Authors critically considered this valid input. The newly modified change was highlighted with a red font color in the cleanly revised main manuscript.

35. Awareness of hypertension-related complications was assessed using a single question with multiple response items. Participants were classified into two categories: Aware: Participants who were knowledgeable about at least two hypertension-related complications. Not Aware: Participants who either did not know of any hypertension-related complications or were aware of only one complication. This assessment was conducted using the Hypertension SelfCare Activity Level Effects (H-SCALE) tool from 9. Tesfaye B,. The authors should reference the following study for the validation and prevalence of hypertension self-care activities: Warren-Findlow J, Seymour RB. Prevalence rates of hypertension self-care activities among African Americans. J Natl Med Assoc. 2011;103(6):503. This reference will provide a robust foundation for the methodology used in measuring self-care activities and awareness in the context of hypertension.

Response: Thank you so much to pointing this out. The reviewer is perfect. We are very grateful for this implicit comment and suggestion. Thank you so much for such a valuable intellectual input. Now, we thoroughly referenced the above mentioned study that means: Warren-Findlow J, Seymour RB. Prevalence rates of hypertension self-care activities among African Americans. J Natl Med Assoc. 2011;103(6):503. In our manuscript for the validation and prevalence of hypertension self-care activities: The newly modified change was highlighted with red font color in the clean revised manuscript.

36. Ethical Considerations Regarding Clinical Variables: Patient Clinical Variables: The manuscript includes patient clinical variables, such as health data related to hypertension. It is crucial for the authors to clarify how they obtained this information. Did the data come from Electronic Health Records (EHR) or Personal Health Records (PHR)? If so, the authors should detail the procedures followed to access this data, ensuring that patient consent and confidentiality were fully protected.

Response: Thanks again for your comment and suggestion. In this case we obtained informed consent from the study participants and also clinical data were obtained from personal medical record. The confidentiality of the data was fully protected. There are no clinical variables that breach of data privacy.

37. Ethical Compliance (PDPA): To avoid potential breaches of data privacy, the authors should ensure that their procedures comply with relevant data protection laws, such as the Personal Data Protection Act (PDPA) or other applicable regulations. Explicit mention of how patient confidentiality was preserved is necessary, including anonymization or de identification of clinical data. The manuscript should also describe how patient consent was obtained for using their clinical data and clarify whether any additional ethical approvals were obtained from institutional review boards (IRBs) or ethics committees.

Response: Thanks again for your comment and suggestion. No need of additional ethical approvals beyond institutional review boards (IRBs) or ethics committees. Because it hasn’t any identifiable information on clinical data. Clinical data mean complication, and any comorbidities of the patient.

38. Discussion and Conclusion (Suggestions for Improvement): The authors have thoroughly discussed the study's characteristics (Table 1, Table 2, and Table 3), along with the determinants of factors associated with uncontrolled hypertension (Table 4). However, the discussion could be improved by ensuring a stronger and more coherent link between the findings presented in the tables and the overarching discussion throughout the manuscript.

Response: Definitely! The reviewer is correct. We are grateful for this implicit comment and suggestion. The authors critically reviewed this vital comment and suggestion as per your robust and critical comments. Authors critically considered this valid input. The newly modified change was highlighted with a red font color in the cleanly revised main manuscript.

39. Study Findings: The key finding of the study is that the odds of having uncontrolled hypertension were 5.21 times greater for those who did not adhere to antihypertensive medication compared to those who did. This important insight should be emphasized more clearly in both the discussion and the conclusion sections.

Response: well thought comments and suggestions!  Thank you so much for such valuable intellectual input. The Author utterly shares the comments and suggestion which immensely enhance the manuscript. The newly modified change was highlighted with red font color in the clean revised manuscript

40. Concise Conclusion: The primary conclusion drawn from the study is that non-adherence to antihypertensive medication is a significant predictor of uncontrolled hypertension. However, while the recommendation to healthcare professionals is appropriate, the conclusion could be extended to propose more comprehensive solutions beyond the current suggestions of weight reduction, health education, and control of comorbidities.

Response: Thank you so much to pointing this out. The reviewer is perfect. We are very grateful for this implicit comment and suggestion. Authors critically considered this valid input. The authors critically reviewed this vital comment and corrected the necessary modification as per your robust and critical comments. The newly modified change was highlighted with red font color in the clean revised main manuscript.

41. Extended Solutions: for instance 1) Improving Medication Adherence: The findings strongly suggest that targeted interventions to improve adherence to antihypertensive medication should be prioritized. 2) This could involve: Implementing structured follow-up and reminder systems for patients.3) Enhancing patient education on the importance of medication adherence and its long-term benefits.4) Utilizing digital health tools, such as mobile apps, to track adherence and offer real-time support.

Response: Absolutely! We really appreciated this important observation. We are at loss for describing your commitments and dedication. Authors critically considered this valid input. Dear respected reviewer, we are grateful for this implicit comment and suggestion. The authors critically reviewed and revised the manuscript. The newly modified change was highlighted with red font color in the clean revised manuscript.

42. Multidisciplinary Care Models: Given the multifactorial nature of hypertension, a team-based care approach involving physicians, nurses, pharmacists, and community health workers should be advocated. This would help to monitor not only blood pressure but also modifiable lifestyle factors more comprehensively.

Response: Absolutely! We really appreciated this important observation. We are at loss for describing your commitments and dedication. Authors critically considered this valid input. Dear respected reviewer, we are grateful for this implicit comment and suggestion. The authors critically reviewed and revised the manuscript. The newly modified change was highlighted with red font color in the clean revised manuscript.

43. Patient-Centered Approaches: Empowering patients to take an active role in managing their hypertension could improve outcomes. This can be achieved by involving them in decision-making regarding their treatment plans and offering personalized care based on individual risk factors.

Response: Thank you, a lot, for pointing out this!  Thank you so much for such valuable intellectual input. The Author utterly shares the comments and suggestion which immensely enhance quality of our manuscript. The newly modified change was highlighted with red font color in the clean revised manuscript.

44. Policy Advocacy: Beyond healthcare professionals, the study suggests that broader community-based and policy-driven initiatives are necessary. This could involve: Implementing national campaigns aimed at increasing awareness about the importance of medication adherence. Introducing financial incentives or subsidies for antihypertensive medications to improve access for low-income populations. By proposing these comprehensive, patient-centered, and policy-driven solutions, the conclusion would not only reflect the study's findings but also offer practical steps that could be taken to address the challenge of uncontrolled hypertension.

Response: Thank you so much to pointing this out. The reviewer is perfect. We are very grateful for this implicit comment and suggestion. Authors critically considered this valid input. The newly modified change was highlighted with red font color in the clean revised manuscript.

Reviewer #2

45. Title: The title is informative but could be more concise. Consider simplifying it to: "Uncontrolled Hypertension in Adult Patients in Addis Ababa Public Hospitals: Prevalence and Associated Factors."

Response: Thank you so much to pointing this out. The reviewer is perfect. We are very grateful for this implicit comment and suggestion. Authors critically considered this valid input. The authors critically reviewed this vital comment and corrected the necessary modification as per your robust and critical comments. The newly modified change was highlighted with red font color in the clean revised main manuscript.

46. Abstract (Lines 15–42): The abstract provides a good summary, but it can be improved by clarifying certain points. For example, instead of saying, "evidence regarding uncontrolled hypertension is very limited," be more specific about the gap in the literature.

Response: Absolutely! We really appreciated this important observation. We are at loss for describing your commitments and dedication. As stated in the manuscript, we specifically addressed the gap in the literature, most of the studies used the previous, Seventh Joint National Committee classification for classifying hypertensive patients into either controlled or uncontrolled, but this study used the eighth JNC. The newly modified change was highlighted with red font color in the clean revised manuscript.

47. I have read many studies conducted on a similar topic in Ethiopia, which indicated prevalence rates of 48.6% in North Ethiopia, 48% in Eastern Ethiopia, 52.7% in Western Ethiopia, and 52.5% in the Tigray region. What distinguishes your research from these studies?

Response: Thank you so much for pointing this out.

- Of course it is true that studies have conducted in Ethiopia regarding uncontrolled hypertension. However, in Addis Ababa Region, which is the Capital City of Ethiopia and the most urbanized city in Ethiopia with different life style from the regions.
- In addition majority of the previously conducted studies in Ethiopia used an outdated, JNC-7 cut-off point for classifying hypertensive patients into either controlled or uncontrolled. The updated, JNC-8 classifications for hypertension control have an updated, revised cut-off points in which our study have utilized.
- Furthermore, the previously conducted studies were conducted in a single hospital (for example, a study conducted at Ayder Comprehensive Specialized Hospital was conducted in one hospital). This increases the chances of generalizability in our study.
- Finally, our study assessed additional variables like knowledge on hypertension self-care activities and complications using the HELM scale and Khat chewing to see if there is association between those variables and uncontrolled hypertension.

. The newly modified change was highlighted with a red font color in the cleanly revised manuscript.

48. Introduction (Lines 45–154): The introduction effectively frames the issue of hypertension, but it could benefit from some tightening. Some statistics could be better integrated into a cohesive narrative. The transition between global and Ethiopian data could also be smoother. There are slight redundancies in defining hypertension and discussing its impact, especially in the first paragraph.

Response: Absolutely! We really appreciated this important observation. We are at loss for describing your commitments and dedication. Authors critically considered this valid input. Dear respected reviewer, we are grateful for this implicit comment and suggestion. The authors critically reviewed, revised and removed redundancies from the section. The newly modified change was highlighted with red font color in the clean revised manuscript.

49. Introduction (Lines 104–150): The reason why you conducted this study was ‘’the review of previous Ethiopian studies mentions inconsistencies.’’ Could you elaborate on what specific factors showed differences across studies? The second main reason you mentioned ‘’No other study conducted on this title in this study area? This is not enough reason to you. Because as I mentioned in question 3 above many studies conducted in Ethiopia. Remove or modify.

Response: Thanks again for your wonderful comment and suggestion. We appreciate the reviewers’ commitment in advancing the quality of the paper. Dear reviewer, we responded the comment of this in question 3 above. The newly modified change was highlighted with red font color in the clean revised manuscript.

50. Study Questions (Lines 155–159).The study questions are clearly stated. However, the second question could be expanded to specify whether the focus is on modifiable factors.

Response: Thank you so much to pointing this out. We appreciate the reviewer comment, however we conducted both on the modifiable and non-modifiable factors with great emphasis were given to modifiable factors. Authors critically considered this valid input. The newly modified change was highlighted with red font color in the clean revised manuscript.

51. Methods - Study Design (Lines 169–230). The study design is well explained, but the sample selection and sampling methods could be clarified further with diagrams or tables for better understanding. The inclusion and exclusion criteria are reasonable, but it would be helpful to briefly mention why pregnant women were excluded (e.g., due to potential pregnancy-related blood pressure changes).

Response: Well thought comments and suggestions! The reviewer is perfect. The authors critically reviewed this vital comment and added the diagram to sample selection and sampling methods for better understanding as per your valuable and critical comments. In addition pregnant mothers were excluded due to potential pregnancy-related blood pressure changes. The newly modified change was highlighted with red font color in the clean revised main manuscript.

52. Sample Size Determination (Lines 182–202). The sample size determination is well explained, but the rationale for choosing specific power and proportion assumptions should be clarified. Suggestion: Consider explaining whether a sensitivity analysis was performed to determine if a different sample size would yield different results.

Response: Thanks a lot for your eagle eyes! The reviewer is correct. We are very grateful for this implicit comment and suggestion. Thank you so much for such valuable intellectual input. Now, based on your suggestions and comments, we have thoroughly revised and sensitivity analysis was performed to determine sample size. The sample size for the study was calculated for both objectives (i.e. for specific objective 1 and for specific objective 2) and the maximum sample size was taken. Proportion assumptions for each variable were taken from reference 27. The newly modified change was highlighted with a red font color in the cleanly revised manuscript.

53. Sampling Procedure (Lines 182–205). Could you clarify how the study handled patients who missed their follow-up visits during the study period?

Response: Thanks a lot for your comment and suggestion. We used patients’ visit as a sampling frame for our study. For instance, we recruited patients based on their visit at hypertension follow-up clinic, based on our inclusion criteria. If we had used patient’s register or patients’ appointment register, those patients who missed their appointment (follow-up) would have been an issue. Otherwise, in our case it is not an issue. The newly modified change was highlighted with a red font color in the cleanly revised manuscript.

54. Data Collection Tools (Lines 205–238). The data collection process is described in detail, but more explanation is needed on why specific scales like H-SCALE and HELM were chosen. The decision to modify the scales should be elaborated to help readers understand the implications of these changes.

Response: Thank you so much for pointing this out. The reviewer is flawless. We greatly appreciate your implied observation and recommendation. The authors include the comments here below. The H-SCALE is preferred because it offers a more comprehensive and detailed evaluation of a patient's status; as it includes medication adherence, physical activity, smoking, salt intake, alcohol, weight management**.** By combining these different aspects, the H-SCALE paints a more complete picture of a patient's hypertension control.

This allows healthcare providers to identify areas of strength and weakness in the patient's management plan. In essence, the H-SCALE offers a more comprehensive and patient-centered approach to assessing hypertension control compared to relying solely on blood pressure readings. This leads to better identification of patients at risk; more targeted interventions, and ultimately, improved outcomes for individuals with hypertension.

In addition, the HELM scale was used to supplement the Knowledge of self-care, life style management and complications of hypertension variable.

Modification of the H-SCALE tool was made to fit into the local context; particularly in DASH diet section where some of the foods are not common in Ethiopian context and including them in the questionnaire is not relevant. For example, a question “Eat processed meats such as ham, bacon, bologna, or sausage?” was excluded from the questionnaire; as these foods are not eaten in Ethiopia. The newly modified change was highlighted with red font color in the clean revised manuscript.

55. Operational Definitions (Lines 252–295). The operational definitions are clear, but certain concepts, such as adherence to medication and diet, could be expanded further to clarify how they were measured in real-time versus self-reported adherence.

Response: Definitely! Thank you very much. We appreciate this important observation. Dear reviewer, you are perfect. We critically revised, addressed and explained this important observation in the revised manuscript. Please refer to the revised newly modified manuscript.

56. Data Quality Management (Lines 314–332). The steps to ensure data quality is mentioned, but there could be more emphasis on minimizing bias. Was inter-rater reliability tested for the nurses collecting the data?

Response: Definitely! The reviewer is correct. We are grateful for this implicit comment and suggestion. The authors critically reviewed this vital comment and corrected the necessary modification as per your robust and critical comments. The newly modified inter-rater reliability tested was highlighted with red font color in the clean revised main manuscript.

57. Results (Lines 345–385). The results are presented clearly. However, the discussion on BMI and age could benefit from deeper contextualization. The high proportion of patients not adhering to medication or diet (51.2% and 79.4%, respectively) warrants further discussion on the reasons for such low adherence.
-Suggestion: Including more visual elements (e.g., charts or figures) would make the results easier to digest.

Response: Absolutely! We really appreciated this important observation. We are at loss for describing your commitments and dedication. Authors critically considered this valid input. Dear respected reviewer, we are grateful for this implicit comment and suggestion. The authors critically reviewed, revised and included charts and figures to the manuscript. The newly modified change was highlighted with red font color in the clean revised manuscript.

58. Discussion (Lines 399–492). The discussion compares the results to several international studies, which is good. However, it would benefit from mentioning how cultural factors, healthcare access, and systemic differences might explain the disparities between Addis Ababa and other global cities. It would also be helpful to explore more deeply why certain factors (e.g., diet non-adherence or the number of antihypertensive medications) are so prevalent in this population.

Response: Response: Well thought comments and suggestions! The reviewer is perfect. The authors critically reviewed this vital comment and corrected the necessary modification as per your valuable and critical comments. The authors compare Addis Ababa with global cities and explore certain factors (e.g., diet non-adherence or the number of antihypertensive medications) in the study. The newly modified change was highlighted with red font color in the clean revised main manuscript.

59. Limitations (Lines 495–502): The study limitations are acknowledged, but they could be expanded. How significant do you think social desirability or recall bias was?

Response: Absolutely! We really appreciated this important observation. We are at loss for describing your commitments and dedication. Authors critically considered this valid input. Dear respected reviewer, we are grateful for this implicit comment and suggestion. The authors critically reviewed revised and expanded social desirability and recall bias. Since most of the H-SCALE questions are self-reported and as participants may tend to respond in a manner that will be viewed favorably by others, social desirability bias might be present. For example, for alcohol drinking the participants might respond as they were alcohol abstainers even if they drank alcohol. Recall bias could also be present as the tool was self-reported and it was dependent on their memory, some recall bias might happen. The newly modified change was highlighted with red font color in the clean revised manuscript.

60. Conclusion (Lines 503–517). The conclusion summarizes the findings well, but it could be more impactful by highlighting the policy implications. The call for "strategies for food vendors to prepare low-salt diets" could be elaborated with more practical recommendations.

Response: Thank you so much for such valuable intellectual input. The reviewer is perfect. We are very grateful for this implicit comment and suggestion. Thank you so much for such valuable intellectual input. The authors critically considered this valid input and include strategies for food vendors in simple and practical way. The newly modified change was highlighted with a red font color in the cleanly revised manuscript.

End of the response

Thank you in advance!!!
