# Supplementary material for: Uncontrolled hypertension among adult hypertensive patients in Addis Ababa public hospitals: A cross-sectional study of prevalence and associated factors
Source: PLoS One. 2024 Dec 30;19(12):e0316461. doi: 10.1371/journal.pone.0316461 (PMC11684623; doi:10.1371/journal.pone.0316461)
Supplement: Attachment — Submitted filename: Response to Reviewers.docx [file pone.0316461.s004.docx]

**Dear editor and reviewer,**

Response: The authors are thankful for the detailed and specific comments on the manuscript. As it is a scientific study, we strongly believe that this will assist to meet high academic standards and ensure that our findings contribute effectively to the existing body of knowledge on uncontrolled hypertension. Most comments focused on the introduction, methodology, result and conclusion section as well as journal requirements. Therefore, detailed and extensive revision is made regarding introduction, methodology, result and conclusion section as well as journal requirements. With all this revision, we believe that the study will meet high academic standards and contribute to existing body of knowledge in the study area. Specific responses and revisions made are provided below and highlighted in the main document. Hopefully, the editor will consider the revised manuscript for publication.

***“Uncontrolled Hypertension in Adult Hypertensive Patients in Addis Ababa Public Hospitals: A cross-sectional study of Prevalence and Associated Factors*.”**

To: editor,

Above all, we thank you for your constructive suggestions and comments on this manuscript that would improve the substance and content of the study. We considered each comment and clarification question of editor on the manuscript thoroughly. Our point-by-point responses for each comment and question are described in detail on the following pages. All newly modified changes were highlighted within the main manuscript. Please see the red font color in the clean revised manuscript.

Editor Comments and Suggestions

1. If you would like to make changes to your financial disclosure, please include your updated statement in your cover letter

Response: Thanks a lot for your suggestions! This revised manuscript has no any change of financial disclosure.

2. Guidelines for resubmitting your figure files are available below the reviewer comments at the end of this letter.

Response: Thanks a lot for your eagle eyes! The editor is correct. We are very grateful for this implicit comment and suggestion. Thank you so much for such valuable intellectual input. Now, based on your suggestions and comments, we have thoroughly, uploaded in to the Preflight Analysis and Conversion Engine (PACE) digital diagnostic tool to change the figure based on the PLOS ONE submission guideline. The newly modified change was highlighted with a red font color in the cleanly revised manuscript.

3. If applicable, we recommend that you deposit your laboratory protocols in [protocols.io](http://protocols.io/) to enhance the reproducibility of your results. Protocols.io assigns your protocol its own identifier (DOI) so that it can be cited independently in the future. Additionally, PLOS ONE offers an option for publishing peer-reviewed Lab Protocol articles, which describe protocols hosted on [protocols.io](http://protocols.io/).

Response: Thank you very much. The editor is perfect, but this study is not laboratory protocol.

Additional Editor Comments:

4. It would be preferable to change your title " Uncontrolled Hypertension among Adult Hypertensive Patients in Addis Ababa Public Hospitals: A cross-sectional study of Prevalence and Associated Factors"

Response: Thanks a lot for your eagle eyes! The editor is correct. We are very grateful for this implicit comment and suggestion. Thank you so much for such valuable intellectual input. Now, based on your suggestions and comments, we have thoroughly modified, edited and revised the title. The newly modified change was highlighted with a red font color in the cleanly revised manuscript.

5. Please rephrase lines 33-34.

Response: Thank you very much. We are grateful for this implicit comment and suggestion. The authors critically reviewed and paraphrased lines 33-34 as per your robust and critical comments. The newly modified change was highlighted with red font color in the clean revised main manuscript.

6. Rephrase and summarize with two pages from lines 47-154.

Response: Thanks a lot for your eagle eyes! The editor is perfect. We are grateful for this implicit comment and suggestion. The authors critically reviewed this vital comment and paraphrased and summarized with in two pages. The newly modified change was highlighted with red font color in the clean revised main manuscript.

7. Line 57: Please remove dot before hypertension.

Response: Thanks a lot for your eagle eyes! The editor is correct. We are very grateful for this implicit comment and suggestion. Now, based on your suggestions and comments, we have removed the dot before the word hypertension. The newly modified change was highlighted with red font color in the clean revised manuscript.

8. Line 58: Please update reference 3 and 4 with the recent Lancet publications on global burden of 288 causes of death and Burden of 88 risk factors..

Response: Definitely! Many thanks for it. We are grateful for this crucial remark. Editor, you are flawless. This significant observation of highlights was thoroughly addressed in the updated paper. The newly modified change was highlighted with red font color in the clean revised manuscript.

9. Please use the complete form once followed by the abbreviation. You have written the complete form of LMICs three times (Lines 67, 87.).

Response: Definitely! Thank you very much. We appreciate this important observation. Dear editor, you are perfect. We made significant revisions on it. Please refer to the revised red font color in the cleanly revised manuscript.

10. Please delete study questions. You have already mentioned them in lines 151 and 152.

Response: Thanks a lot for your eagle eyes! The editor is correct. We are very grateful for this implicit comment and suggestion. Thank you so much for such valuable intellectual input. Now, based on your suggestions and comments, we have deleted the study questions from the manuscript. The newly modified change was highlighted with a red font color in the cleanly revised manuscript.

11. Please make Figure 1 clearer. Please remove the green color..

Response: Thank you so much for pointing this out! Many thanks for it. We are grateful for this crucial remark. Editor, you are flawless. This significant observation was thoroughly addressed. Please refer to the neatly corrected figure and the updated red font color.

12. In operational definitions; Provide details what was the definition for medical comorbidities in the study.

Response: Definitely! Thank you very much. We are very grateful for this implicit comment and suggestion. Thank you so much for such valuable intellectual input. Now, based on your suggestions and comments, we have thoroughly, revised, paraphrased and edited the manuscript. The newly modified change was highlighted with red font color in the clean revised manuscript.

13. Figure 2. Please delete figure legend from inside the figure and also follow PLOS ONE's figure requirements

Response: Thank you so much for such valuable intellectual input. The editor is perfect. We are very grateful for this implicit comment and suggestion. Thank you so much for such valuable intellectual input. The authors critically considered this valid input and revised it. The newly modified change was highlighted with a red font color in the cleanly revised manuscript.

14. Figure 3. Please delete figure legend from inside the figure. The pie chart can be further improved. Please look at the paper for reference.

Response: Thank you, a lot, for pointing out this!  I am really grateful for your insightful comments. The author fully offers the utterly shares the comments and suggestion, which significantly improve the quality of figures. The newly modified change was highlighted with red font color in the clean revised manuscript.

15. Figure 4. Please delete figure legend from inside the figure. The pie chart can be further improved..

Response: Thank you so much for pointing this out. The editor is flawless. We greatly appreciate your implied observation and recommendation. We critically revised and addressed this important observation in the revised manuscript. The newly modified change was highlighted with red font color in the clean revised manuscript.

16. The conclusion section is redundant, and could be condensed to focus on the key findings and implications (not more than a half page)..

Response: Definitely! The editor is correct. We are grateful for this implicit comment and suggestion. The authors critically reviewed and removed redundant sections summarized in half pages. The newly modified change was highlighted with red font color in the clean revised manuscript.

17. The language and the flow of the writing should be improved.

Response: Response: Thank you so much to pointing this out. The reviewer is perfect. We are very grateful for this implicit comment and suggestion. Authors critically considered this valid input. The authors critically reviewed this vital comment and corrected the necessary modification as per your robust and critical comments. The newly modified change was highlighted with red font color in the clean revised main manuscript

**To Reviewer**

Above all, we thank you for your constructive comments and helpful suggestions that helped us to improve and enrich our study. We considered each comment and clarification question of the reviewer on the manuscript thoroughly. Our point-by-point responses for each comment and question are described in detail on the following pages. All newly modified changes were highlighted with red font color in the main manuscript. Please see the red font color in the clean revised manuscript.

Reviewer #1

18. Thank you for the opportunity to review this revised manuscript titled "Uncontrolled Hypertension in Adult Patients in Addis Ababa Public Hospitals: Prevalence and Associated Factors." I appreciate the authors’ responsiveness and diligence in addressing all previous comments.

Response: Thank you so much! Many thanks for it. The authors would also thankful for your effort and unwavering dedication to improve the quality of this study.

19. The authors have thoroughly addressed each suggestion, resulting in a clearer and more comprehensive presentation of the study. I am pleased with the revisions, which have strengthened the manuscript, and I have some of comments to considerate below.

Response: Thank you so much. .We are very grateful for the comment and suggestion you provide. Authors critically considered your valid input. The authors critically reviewed your vital comment and corrected the necessary modification as per your robust and critical comments. Thank you again for your comment to improve the quality of the manuscript.

20. The title change enhances clarity and aligns well with the manuscript's focus.

Response: Thank you so much for such valuable intellectual input. The reviewer is perfect. We are very grateful for this implicit comment and suggestion. Thank you so much for such valuable intellectual input. The authors critically considered this valid input. The newly modified change was highlighted with a red font color in the cleanly revised manuscript.

21. The abstract is significantly improved. However, the conclusion could better

Emphasize the most critical risk factors for uncontrolled hypertension in this

setting. Key factors to highlight include non-adherence to antihypertensive

medications (OR 5.21), use of multiple medications (OR 3.10), lack of physical

exercise (OR 2.84), and low adherence to low-salt and DASH diets (OR 2.74). To

address these, focus on enhancing patient education on medication adherence,

promoting lifestyle changes, and leveraging digital health tools, like mobile apps,

for real-time support and adherence tracking.

Response: Thanks a lot for your eagle eyes!  The reviewer is correct. We are very grateful for this implicit comment and suggestion. Thank you so much for such a valuable intellectual input. The authors critically considered this valid input. The newly modified change was highlighted with a red font color in the cleanly revised manuscript.

22. Thank you for the thoughtful revisions. The background, methods, results, and

discussion sections are much improved and offer a clearer presentation of the

study compared to the previous version.

Response: Thank you so much! Many thanks for it. The authors would also thankful for your effort and unwavering dedication to improve the quality of this study

23. However, the conclusion remains overly lengthy. I recommend condensing and

refining it to ensure a more concise and impactful summary of the key findings

and recommendations.

Response: Thank you so much for pointing this out. The reviewer is flawless. We greatly appreciate your implied observation and recommendation. The authors summarize, condense, refine and revised the conclusion and recommendation section. The newly modified change was highlighted with red font color in the clean revised manuscript.

24. From the previous comments about Recommendations include implementing

Follow-up and reminder systems, enhancing patient education on adherence

benefits, and using digital health tools to support real-time tracking..

Response: Definitely! Thank you very much. We appreciate this important observation. Dear reviewer, you are perfect. We critically revised and addressed this important observation in the revised manuscript. Please refer to the revised newly modified manuscript.

25. Also in line 628 -640 are the recommendation as a Multi-disciplinary Care

Models, Patient-Centered Approaches, Policy Advocacy, the authors included in

this part and might make it concise and decrease the duplicate in line 641-649.

Response: Thank you, a lot, for pointing out this!  Thank you so much for such valuable intellectual input. The Author utterly shares the comments and suggestion which immensely enhance the manuscript. The newly modified change was highlighted with red font color in the clean revised manuscript.

26. It would be beneficial to delete lines 651-657, as they introduce additional

recommendations that do not directly align with the main focus of the study's

findings or with the core recommendations on improving hypertension control.

This section introduces new topics, such as mass-sporting activities and specific

local projects, which may dilute the impact of the more targeted suggestions on

medication adherence, lifestyle changes, and digital tools. Additionally, the

recommendation for future studies, though useful, may not be necessary in the

conclusion if it has already been mentioned in the discussion.

Response: Thank you so much to pointing this out. The reviewer is perfect. We are very grateful for this implicit comment and suggestion. Authors critically considered this valid input. Dear respected reviewer, we removed mass-sporting activities and specific local projects from the manuscript. The newly modified change was highlighted with red font color in the clean revised manuscript .

27. In summary, removing these lines would help make the conclusion more concise and focused, allowing the key findings and primary recommendations to standout more clearly.

Response: Thank you very much. The reviewer is perfect. We are grateful for your robust and rigorous comments and suggestions. Hence, the author utterly shares the concerns and comments of the reviewer. Thus, now, with great appreciation, the authors have considered your valid concern and correct it as per your comment and suggestion. The newly modified change was highlighted with red font color in the clean revised manuscript.

End of the response

Thank you in advance!!!
