# Supplementary material for: Uncontrolled hypertension among adult hypertensive patients in Addis Ababa public hospitals: A cross-sectional study of prevalence and associated factors
Source: PLoS One. 2024 Dec 30;19(12):e0316461. doi: 10.1371/journal.pone.0316461 (PMC11684623; doi:10.1371/journal.pone.0316461)
Supplement: Attachment — Submitted filename: Response to Reviewers.docx [file pone.0316461.s005.docx]

**Dear editor and reviewer,**

Response: The authors are thankful for the detailed and specific comments on the manuscript. As it is a scientific study, we strongly believe that this will assist to meet high academic standards and ensure that our findings contribute effectively to the existing body of knowledge on uncontrolled hypertension. Most comments focused on methodology, figure, language and grammar. Therefore, revision is made on methodology, figure, language and grammar. With all this revision, we believe that the study will meet high academic standards and contribute to existing body of knowledge in the study area. Specific responses and revisions made are provided below and highlighted in the main document. Hopefully, the editor will consider the revised manuscript for publication.

***“Uncontrolled Hypertension among Adult Hypertensive Patients in Addis Ababa Public Hospitals: A cross-sectional Study of Prevalence and Associated Factors*.”**

To: editor,

Above all, we thank you for your constructive suggestions and comments on this manuscript that would improve the substance and content of the study. We considered each comment and clarification question of editor on the manuscript thoroughly. Our point-by-point responses for each comment and question are described in detail on the following pages. All newly modified changes were highlighted within the main manuscript. Please see the red font color in the clean revised manuscript.

Editor Comments and Suggestions

1. If you would like to make changes to your financial disclosure, please include your updated statement in your cover letter

Response: Thanks a lot for your suggestions! This revised manuscript has no any change of financial disclosure.

2. Guidelines for resubmitting your figure files are available below the reviewer comments at the end of this letter.

Response: Thanks a lot for your comment! The editor is correct. we were already uploaded in to the Preflight Analysis and Conversion Engine (PACE) digital diagnostic tool to change the figure based on the PLOS ONE submission guideline in the previous submission. Now no need of uploading the file again, since it is correct.

3. If applicable, we recommend that you deposit your laboratory protocols in [protocols.io](http://protocols.io/) to enhance the reproducibility of your results. Protocols.io assigns your protocol its own identifier (DOI) so that it can be cited independently in the future. Additionally, PLOS ONE offers an option for publishing peer-reviewed Lab Protocol articles, which describe protocols hosted on [protocols.io](http://protocols.io/).

Response: Thank you very much. The editor is perfect, but this study is not laboratory protocol.

Journal Requirements:

4. Please review your reference list to ensure that it is complete and correct. If you have cited papers that have been retracted, please include the rationale for doing so in the manuscript text, or remove these references and replace them with relevant current references. Any changes to the reference list should be mentioned in the rebuttal letter that accompanies your revised manuscript. If you need to cite a retracted article, indicate the article’s retracted status in the References list and also include a citation and full reference for the retraction notice.

Response: Thank you very much. We are grateful for this implicit comment and suggestion. We are not cited retracted article in the manuscript and no any changes have been made on reference list of the manuscript.

Additional Editor Comments:

5. Uncontrolled hypertension: How was outcome defined? What about patients who do not have DM and chronic kidney diseases but they were taking AHMs.

Response: Thanks a lot for your eagle eyes! The editor is correct. We are very grateful for this implicit comment and suggestion. Thank you so much for such valuable intellectual input. Now, based on your suggestions and comments, we have thoroughly modified, edited and revised. The newly modified change was highlighted with a red font color in the cleanly revised manuscript.

6. I do not understand what the authors are explaining from lines 245-266. The author should only provide details for their method of measuring adherence.

Response: Thank you very much. We are grateful for this implicit comment and suggestion. The authors critically reviewed and paraphrased lines 245-266 and explain it in clear manner. The newly modified change was highlighted with red font color in the clean revised main manuscript.

7. Please delete figures 2 and 3. These data are available in Tables.

Response: Thank you so much for pointing this out! Many thanks for it. We are grateful for this crucial remark. Editor, you are flawless. This significant observation was thoroughly addressed. Please refer to the neatly corrected and the updated manuscript.

8. Please revise in-text citations. Please use []. Moreover, please merge citations when you are using more than one citation.

Response: Thanks a lot for your eagle eyes! The editor is correct. We are very grateful for this implicit comment and suggestion. Now, based on your suggestions and comments, we have corrected text citation. The newly modified change was highlighted with red font color in the clean revised manuscript.

9. The font in Tables and text is different.

Response: Definitely! Many thanks for it. We are grateful for this crucial remark. Editor, you are flawless. This significant observation of highlights was thoroughly addressed in the updated paper and corrected the font size of the table. The newly modified change was highlighted with red font color in the clean revised manuscript.

10. The article needs to be corrected for language and grammar. There are still many capitalization and punctuation errors

Response: Definitely! Thank you very much. We appreciate this important observation. Dear editor, you are perfect. We made significant revisions on it. Please refer to the revised red font color in the cleanly revised manuscript.

End of the response

Thank you in advance!!!
